# The Addition of a Pheromone to a Floral Lure Increases Catches of Females of the Click Beetle *Agriotes ustulatus* (Schaller) (Coleoptera: Elateridae)

**DOI:** 10.1007/s10886-019-01087-z

**Published:** 2019-07-16

**Authors:** Miklós Tóth, Lorenzo Furlan, István Szarukán, Antal Nagy, József Vuts, Teodora Toshova, Dimitar Velchev, Zsófia Lohonyai, Zoltán Imrei

**Affiliations:** 1 Plant Protection Institute, Centre for Agricultural Research, Hungarian Academy of Sciences, Herman O. u. 15, Budapest, H-1022 Hungary; 2 Veneto Agricoltura, Settore Ricerca Agraria, I-35020 Legnaro, Italy; 3 Institute of Plant Protection, Univesity of Debrecen, POB 400, Debrecen, H-4002 Hungary; 4 Present Address: Department of Biointeractions and Crop Protection, Rothamsted Research, Harpenden, Hertfordshire, AL5 2JQ UK; 5 Institute of Biodiversity and Ecosystem Research, Bulgarian Academy of Sciences, blvd. Tsar Osvoboditel 1, BG-1000 Sofia, Bulgaria; 6 Maize Research Institute, BG-5835 Knezha, Bulgaria

**Keywords:** Bisexual lure, (*E*)-anethol, (*E*)-cinnamaldehyde, IPM, Floral volatile

## Abstract

*Agriotes ustulatus* is an economically important click beetle in Europe. A female-produced pheromone, (*E,E*)-farnesyl acetate, has been identified and is used for monitoring and detecting males. More recently, a floral lure targeting females with modest, but significant, activity has been described. Based on preliminary data, we hypothesized, that similar to the effects on the congeneric *A. brevis*, addition of the pheromone to the floral lure should improve female *A. ustulatus* catches. Also, as click beetles have been reported to respond to white light, we studied possible interactions between visual and chemical cues. In field trials, the addition of the synthetic pheromone to the floral lure resulted in a dramatic increase in the number of females trapped, whereas male catches remained unaffected and equal to those in traps baited with pheromone only. A white visual cue did not influence trap catches. Maximum catches of both sexes of *A. ustulatus* can be achieved using the pheromone and the floral lure inside the same trap. Furthermore, the compounds can be formulated in a single polyethylene bag dispenser, making handling of the trap easier. Due to a much larger proportion of females in the catch, this improved trap may be a promising tool for semiochemical-based, environmentally sound agricultural practice against this important pest.

## Introduction

The Elateridae, or click beetles, are an economically important beetle family, the larvae of which are known as wireworms and damage a wide variety of crops. *Agriotes ustulatus* (Schaller, 1783) is considered to be one of the most destructive representatives of the Elateridae in Europe (Ritter et al. 2014), Central and Western Asia, and North Africa (Čačija et al. 2018; Furlan 1996, 2014; Furlan and Toffanin 1996).

Initial studies aiming to develop an attractant for the detection and monitoring of *A. ustulatus* identified (*E,E*)-farnesyl acetate as the major component in female pheromone gland extracts, together with several related compounds as minor constituents (Kudryavtsev et al. 1993; Siirde et al. 1993; Yatsynin and Lebedeva 1984; Yatsynin et al. 1996). Later field tests established that none of the minor components influenced the activity of (*E,E*)-farnesyl acetate (Tóth et al. 2003) which, on its own, is highly attractive to male *A. ustulatus* in many European countries (Furlan et al. 2007).

Efforts to develop a female-targeted lure commenced soon after identification of the pheromone, because a trapping system that also captures females is potentially more useful for plant protection. As a result, a combination of the floral compounds (*E*)-anethol and (*E*)-cinnamaldehyde was found to be a lure for adults of *A. ustulatus* (Tóth et al. 2011). When placed in traps, this bait attracted significant numbers of beetles, a high percentage of which were female.

Originally, it was considered that click beetles have a “classical” type of sex pheromone system, with females producing and only males responding to the signal (Siirde et al. 1993; Yatsynin et al. 1996). However, it was surprising that the synthetic pheromones of *A. sordidus* (Illiger, 1807) (Tóth et al. 2015) and *A. brevis* (Candeze, 1863) (Vuts et al. 2018) also attracted females, albeit in much smaller numbers than males. We also observed this phenomenon with *A. ustulatus* (L. Furlan and I. Szarukán, unpubl.).

In other beetle families, the occurrence of aggregation pheromones (those produced by one sex but attractive to both) is common; in these cases, the presence of both pheromone and host-plant derived chemicals can result in catches of large numbers of both sexes (Francke and Dettner 2005; Vuts et al. 2014b). Therefore, we hypothesized that this may also be the case with *A. ustulatus*, and conducted research to determine a possible interaction between the pheromone and floral attractant lures. The aim was to develop a potent combined lure, capable of attracting high numbers of both females and males, which can be used by growers in agricultural systems.

## Methods and Materials

Funnel traps (CSALOMON® VARb3) produced by the Plant Protection Institute, Centre for Agricultural Research, Hungarian Academy of Sciences (Budapest, Hungary) were used for the field trials (Imrei et al. 2001; Schmera et al. 2004). These generally had transparent upper funnels, although in some experiments funnels were white (see descriptions of single experiments). For the reflectance spectrum of the white funnels, refer to Schmera et al. (2004). Photos of VARb3 traps can be viewed at *www.csalomontraps.com*. Traps were set at soil level. To kill captured insects, a small piece (1 × 1 cm) of a household anti-moth insecticide strip (Vaportape®, Hercon Environmental Inc., Emigsville, PA, USA) was placed into each catch container.

For the binary floral lure, synthetic (*E*)-anethol and (*E*)-cinnamaldehyde were obtained from Sigma-Aldrich Kft. (Budapest, Hungary). Both compounds were stated by the supplier to be >95% pure. Lures were formulated by loading 100 μl of each compound [ca. 99.8 mg (*E*)-anethol and 105 mg (*E*)-cinnamaldehyde] onto a single 1 cm piece of dental roll (Celluron, Paul Hartmann Ag., Heidenheim, Germany), which was placed in a polyethylene (PE) bag (1.0 × 1.5 cm) made of 0.02 mm linear polyethylene foil, as described in Tóth et al. (2011). The PE dispensers were heat-sealed and the lures wrapped singly in aluminum foil and stored at −18 °C until use. In the field, lures were changed at 3–4 week intervals, as previous experience with similar baits had shown that they start to lose activity after this time (Tóth et al. 2011). The pheromone dispensers were made by loading 50 μl (ca. 45.5 mg) of >95% pure (*E,E*)-Farnesyl acetate (purchased from Sigma-Aldrich Kft., Budapest, Hungary) into PE vial dispensers (Tóth et al. 2003).

Field trapping was conducted in maize fields at several sites in Hungary, Italy and Bulgaria, using accepted methods for trapping experiments (Roelofs and Cardé 1977). Traps were arranged in blocks so that each block contained one trap of each treatment. Traps within blocks were separated by 8–10 m, and blocks were sited at least 30 m apart. The traps were inspected on a regular basis (usually twice weekly), whereupon captured insects were recorded and removed. All *A. ustulatus* specimens caught were sexed.

Catch data were summed for each trap over the duration of the experiment. As the data did not fulfil the requirements for parametric analysis, they were analyzed by the Kruskal-Wallis test. In cases when the Kruskal-Wallis test yielded significance (*P* < 0.05), paired comparisons of treatments were undertaken using the Mann-Whitney U test. All statistical procedures were conducted using the StatView® v4.01 software package (Abacus Concepts, Inc., Berkeley, CA, USA).

### Experiments

#### Experiment 1

This test was run in parallel at two sites: A) Látókép, Debrecen, Hungary (47°31′47.91“ N; 21°38’21.69” E), June 19–August 3, 2007, and B) Moizzi, Eraclea, Italy (45°37′58.48“ N; 12°40’16.19” E), June 11–August 17, 2007. At both sites, five blocks of traps were set up. Treatments included the pheromone (PE vial) or the floral lure (PE bag) alone, and in combination (two lures in one trap). All three treatments were placed in traps with white upper funnels.

#### Experiment 2

This experiment was run to confirm the results obtained in Experiment 1, and to test the effect of white funnels on catches. The test was again run in parallel at the two previous sites: A) at Látókép, June 10–July 25, 2008, and B) at Moizzi, July 11–August 13, 2008. The experimental set up was the same as for Experiment 1, with the exception that all three treatments were tested in traps with white or transparent upper funnels.

#### Experiment 3

To test the effect of increasing doses of (*E,E*)-farnesyl acetate added to the binary floral lure, we ran an experiment in parallel at two sites: A) Stretti, Eraclea, Italy, (45°62′35.48“ N; 12°66’91.07” E), July 9–August 16, 2009, and B) Knezha, Bulgaria, (43°28′48.85“ N; 24°3’22.03” E), June 26–August 11, 2009. Both sites comprised six blocks of traps, and treatments included the floral lure alone (PE bag), the floral lure plus 0.5, 5.0 or 50.0 μl of (*E.E*)-farnesyl acetate (in PE vials), and pheromone [50.0 μl (*E,E*)-farnesyl acetate in PE vials]. All treatments were set in traps with transparent upper funnels.

#### Experiment 4

To determine whether the active ingredients of the binary floral lure and the pheromone could be formulated in a single dispenser without loss of activity, we ran a test in parallel at two sites, each with four blocks of traps: A) Kápolnásnyék, Hungary, (47°23′88.55“ N; 18°67’35.46” E), June 16–July 13, 2018, and B) Vizsoly, Hungary, (48°38′53.30“ N; 21°21’61.70” E), June 10–August 25, 2018. Treatments included: (i) (*E*)-anethol and (*E*)-cinnamaldehyde (100 μl of each) plus 50 μl of (*E,E*)-farnesyl acetate in a single PE vial or PE bag dispenser, (ii) the floral lure (PE bag) and the pheromone (PE vial) in combination (two lures in the same trap), and (iii) unbaited control traps. All treatments were set up in traps with transparent upper funnels.

## Results

### Experiment 1

**Fig. 1:**
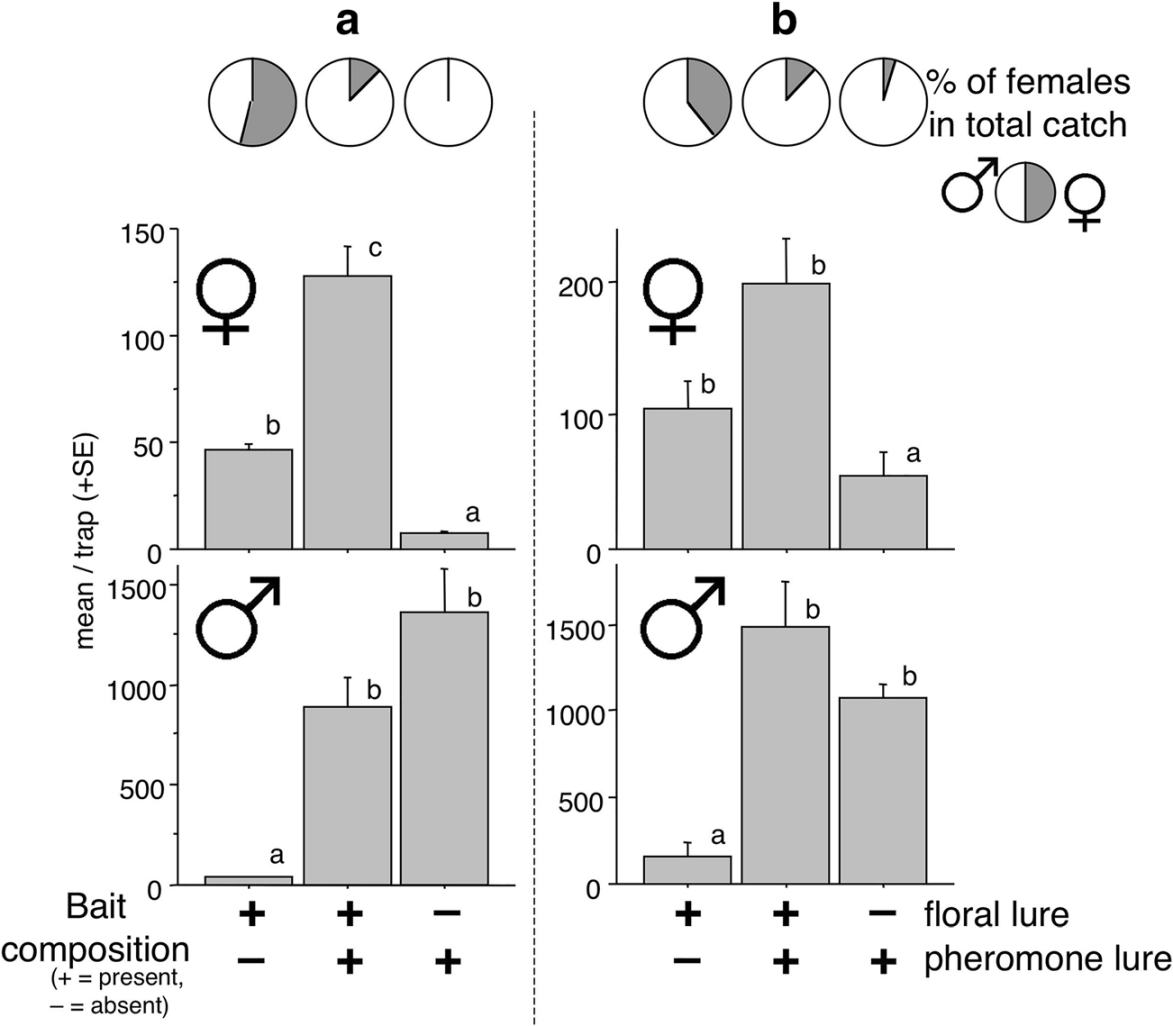
Experiment 1. Mean catches of *Agriotes ustulatus* in traps baited with synthetic pheromone, a binary floral lure, or both lures together. **a** = Trial conducted in Debrecen, Hungary, with total catches of 906 females and 11454 males. **b** = Trial conducted in Moizzi, Italy with total catches of 1794 females and 13632 males. SEMs are given. Bars with the same letter atop are not significantly different (Kruskal-Wallis test, followed by Mann-Whitney U test, α = 0.05)

The distributions of catches among treatments at both sites were almost identical (Fig. 1). The lowest numbers of females were caught in traps with the pheromone only, with more caught in traps with the floral lure. The greatest overall catches were in traps baited with the combination of floral and pheromone lures, with significantly greater catches than in traps baited with the floral lure alone at site A. Traps with the floral lure caught the lowest number of males, in contrast to the high catches in traps with both treatments containing the pheromone lure (Fig. 1).

The percentage of females caught at both sites was lowest in traps with pheromone only and greatest in traps with the floral lure only (Fig. 1). Traps containing both lures showed an intermediate proportion of females.

### Experiment 2

**Fig. 2:**
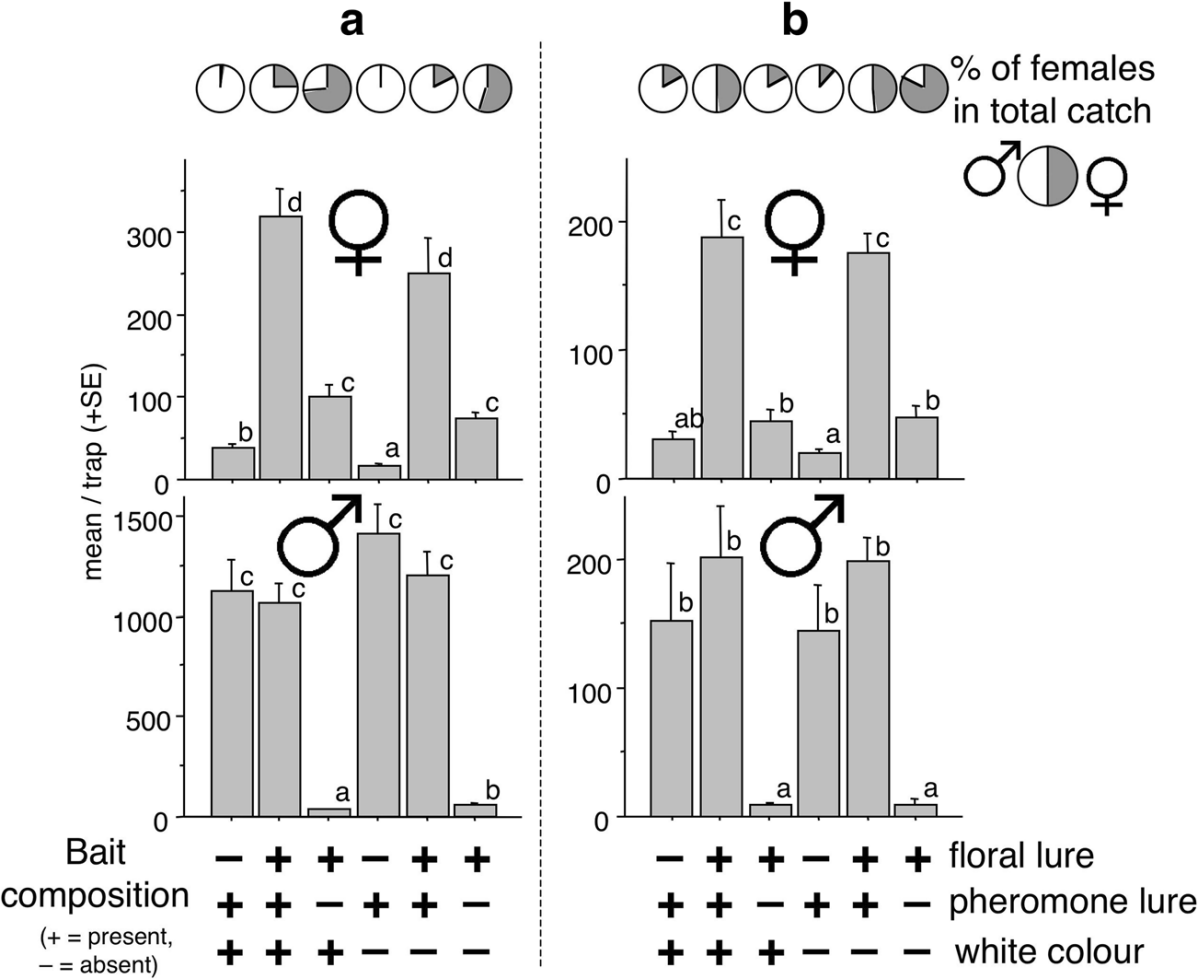
Experiment 2. Mean catches of *Agriotes ustulatus* in white or transparent traps baited with synthetic pheromone, a binary floral lure, or both lures together. **a** = Trial conducted in Debrecen, Hungary with total catches of 4814 females and 29511 males. **b** = Trial conducted in Moizzi, Italy with total catches of 2632 females and 3591 males. SEMs are given. Bars with the same letter atop are not significantly different (Kruskal-Wallis test, followed by Mann-Whitney U test, α = 0.05)

Catches showed a similar trend as for Experiment 1, with the lowest number of females caught in traps with only the pheromone. Significantly more beetles were caught in traps with the floral lure, and the highest catch in traps was with the combination of lures (Fig. 2). The white upper funnel did not generally influence catches, with the exception of more females (relatively low numbers) being caught in traps with white *versus* clear upper funnels baited with the pheromone only (Fig. 2a).

Large numbers of males were caught in traps baited with the pheromone only or with combined lures (these did not differ), while lower catches of males were recorded in traps with the floral lure only (Fig. 2). The white upper funnel did not influence male catches, with the exception of slightly more (but very low) numbers of males caught in traps with the white *versus* clear upper funnels when baited with the floral lure only (Fig. 2a).

Similar to results in Experiment 1, the lowest of females at each site was recorded in traps with the pheromone only. This percentage increased in traps with both lures, with the highest percentage trapped with the floral lure only (Fig. 1).

### Experiment 3

**Fig. 3:**
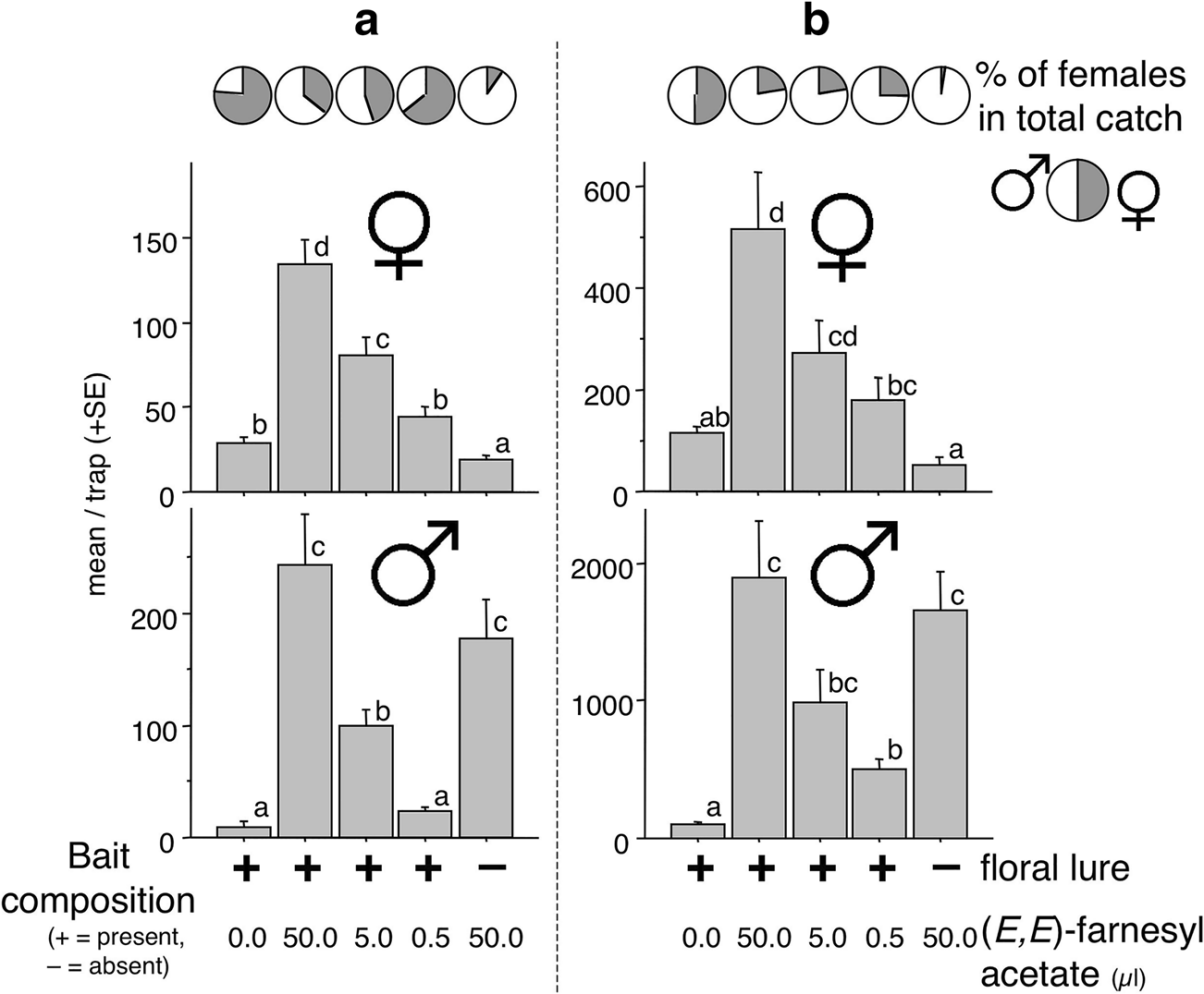
Experiment 3. Mean catches of *Agriotes ustulatus* in traps baited with synthetic pheromone, a binary floral lure, or floral lure plus synthetic pheromone at different doses. **a** = Trial conducted in Stretti, Italy with a total catch of 1850 females and 3331 males. **b** = Trial conducted in Knezha, Bulgaria with a total catch of 30,955 females and 6842 males. SEMs are given. Bars with the same letter atop are not significantly different (Kruskal-Wallis test, followed by Mann-Whitney U test, α = 0.05)

The distribution of catches among treatments at the two sites was similar (Fig. 3). Increased female catches were observed with increasing doses of (*E,E*)-farnesyl acetate, with traps baited with doses above 5 μl catching significantly more females than traps with the floral lure alone. The lowest numbers of females were caught in traps with 50 μl of (*E,E*)-farnesyl acetate by itself.

Male catches were also influenced by (*E,E*)-farnesyl acetate (Fig. 3), with increasing doses resulting in greater catches of males. At site B, catches in the traps containing 5 μl of (*E,E*)-farnesyl acetate were not different than those than in traps with a 50 μl dose. The smallest catches were recorded in traps with the floral lure alone.

The total percentage of females caught showed a similar tendency to the previous experiments, with the highest percentage in traps with the floral lure only, the lowest in traps with only the pheromone lure, and intermediate percentages in traps with floral lure and different amounts of (*E,E*)-farnesyl acetate (Fig. 3).

### Experiment 4

**Fig. 4:**
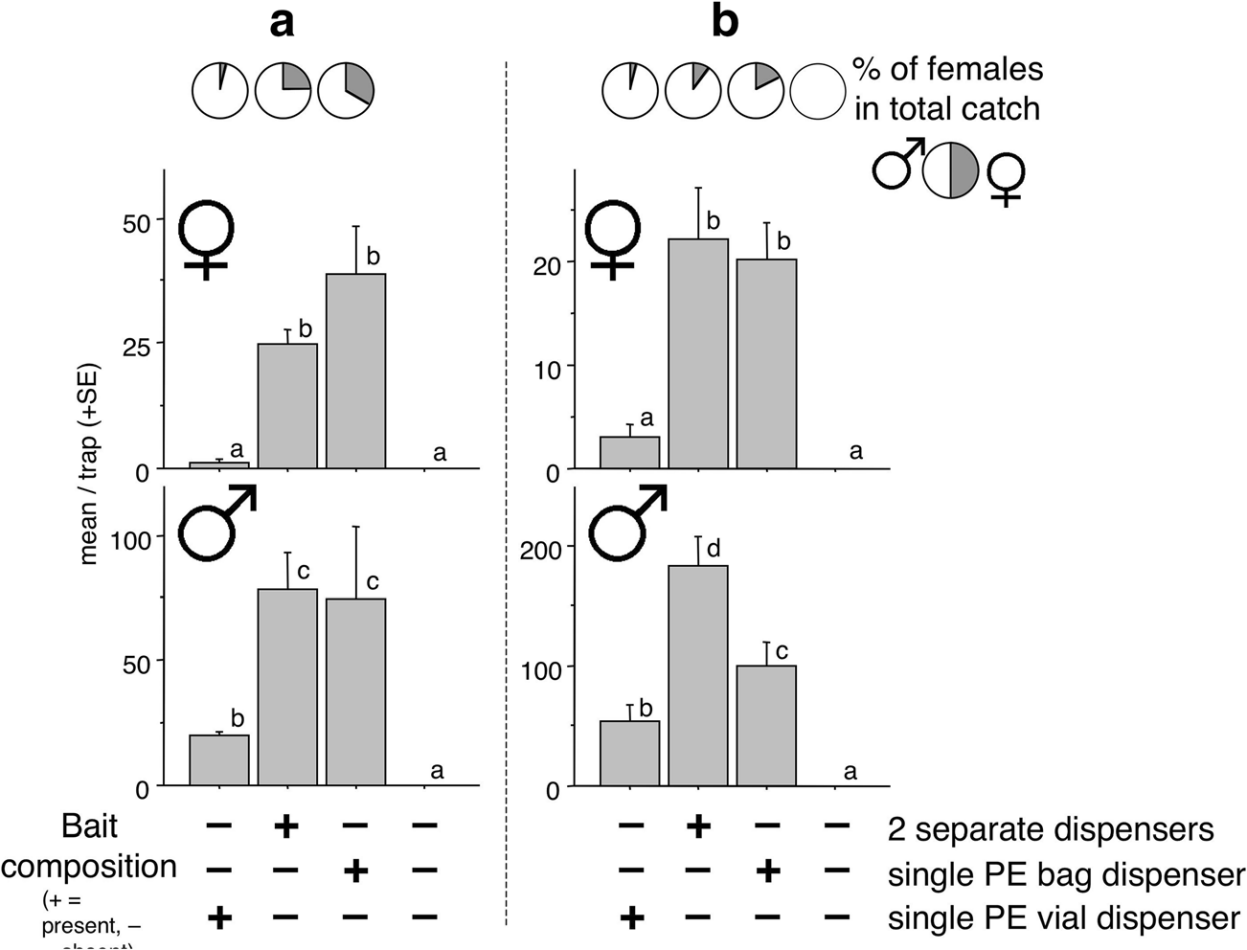
Experiment 4. Mean catches of *Agriotes ustulatus* in traps baited with synthetic pheromone and a floral lure formulated either in separate dispensers or combined in a single PE vial or PE bag. **a** = Trial conducted in Kápolnásnyék, Hungary with a total catch of 259 females and 693 males. **b** = Trial conducted in Vizsoly, Hungary with a total catch of 182 females and 1351 males. SEMs are given. Bars with the same letter atop are not significantly different (Kruskal-Wallis test, followed by Mann-Whitney U test, α = 0.05)

Both sites had similar results. The greatest female catches were in traps with separate dispensers or with a single PE bag dispenser, not differing from each other (Fig. 4). Significantly lower numbers were caught in traps with the single PE vial dispenser, not different from the zero catch of unbaited traps.

The distribution of male catches was very similar to that of females, with the greatest numbers caught in traps with two separate dispensers or with a single PE bag dispenser (Fig. 4). Significantly lower catches were recorded in traps with the single PE vial dispenser; however, these were greater than catches in unbaited traps.

The lowest percentage of females was in traps with a single PE vial dispenser, with greater percentages caught in traps with a single PE bag or with two dispensers (Fig. 4). At one site, a single male was found in an unbaited trap (Fig. 4b).

## Discussion

Various interactions between host-plant chemicals and insect pheromones are known, including host-plant influence on pheromone production and release, enhancement or synergism of attraction to pheromones by host odor, interruption of response to pheromones through inhibitory or repellent effects of plant volatiles, and attraction of a herbivore’s natural enemies to feeding damage (Landolt and Phillips 1997; Reddy and Guerrero 2004).

Our experiments demonstrated that catches of female *A. ustulatus* in traps baited with a binary floral lure were increased by the addition of a synthetic pheromone. The dose-dependent response of females to increasing amounts of (*E,E*)-farnesyl acetate (the active constituent in the pheromone lure) supports the notion that the pheromone is responsible for this phenomenon. In contrast, catches of males in traps baited with the pheromone were not influenced by the presence of the floral lure.

To our knowledge, the only click beetle species for which increased activity to the combined presence of a synthetic pheromone and a plant-derived attractant has been demonstrated is the congeneric *A. brevis* (Vuts et al. 2014a). However, in that study the origin of the plant compounds was from cut, not intact, plant material and its relationship to the biology of the insect is unknown. By contrast, the effect addressed by our *A. ustulatus* study likely mimics the chemicals emitted by intact flowers that are utilized as food sources (nectar and pollen) by adults (Tóth et al. 2011). However, we note that the quantities and ratios of the different compounds tested here were used primarily to maximize trap catch and their relationship to the actual quantities and ratios of odors encountered in nature is not known. Further studies are needed to determine the relevance of these actual odors to the biology of *A. ustulatus*.

An effective trap design should incorporate several types of attractive cues for the target pest. Often, the effects of chemical attractants can be enhanced by the addition of visual cues, because insects often use visual stimuli to locate a host (Raguso 2004). Both sexes of adult *A. ustulatus* are diurnal and visit a range of white flowers (e.g., species of *Convolvulus, Daucus*, etc.) to feed (Furlan 1996). However, in our study, the use of white, as opposed to clear, funnels in the baited traps had little or no effect on trap catch, suggesting the predominance of the chemical cues. This is consistent with earlier findings of white traps being similarly ineffective when presented with a floral lure (Tóth et al. 2011).

When monitoring *A. ustulatus* using a trap baited with both floral and pheromone components, it is likely more advantageous for the trap to catch large numbers of females rather than males, as this should provide more reliable data on the timing of oviposition, which, in turn could result in more precise pest-control decisions (Wall 1989; Witzgall et al. 2010). In fact, all practical approaches using pheromones for attracting males (mass trapping, lure and kill, etc.) are also likely to be enhanced through additional strong attraction of female insects.

For practical purposes, catches of both sexes of *A. ustulatus* can be increased if traps contain both floral and pheromone lures in a single PE bag dispenser. Use of this combined lure is not only simpler than using separate lures/traps, but it could also improve monitoring, as well as direct control (e.g., mass trapping, lure and kill, etc.) (Furlan and Kreutzweiser 2015; Furlan et al. 2018) methods for this pest in integrated management systems.
